# Vulnerability of Rural Households to Climate Change and Food Insecurity in Enebse Sar Midir District, Amhara Region, Ethiopia

**DOI:** 10.1155/tswj/7934040

**Published:** 2026-05-14

**Authors:** Kiros Getachew Belachew, Berhanu Tadesse Beyene, Balew Yibeltal Bezabih

**Affiliations:** ^1^ College of Agriculture and Natural Resources, Debre Markos University, Debre Markos, Ethiopia, dmu.edu.et

**Keywords:** climate adaptation strategies, climate change, food security, household food insecurity

## Abstract

Climate change poses profound global challenges, especially for agriculture and food security in developing countries. This study investigates the impact of climate change on household food security and assesses the effectiveness of farm‐level adaptation strategies in mitigating its impacts in the Enebse Sar Midir District of the East Gojjam Zone, Ethiopia. Data were collected through a household survey of 184 rural households using structured questionnaires and analyzed using SPSS Version 26. The findings reveal that 85.9% of respondents observed changes in temperature, while 90.2% noted altered rainfall patterns. The key climate‐related challenges affecting food security included drought (79.3%), erratic rainfall, and flooding. Household food security was assessed using indicators such as the months of adequate household food provisioning (MAHFP), Household Food Insecurity Access Scale (HFIAS), and household dietary diversity score (HDDS). The study showed that 33.7%, 42.9%, and 32.1% of households were food secure according to MAHFP, HFIAS, and HDDS, respectively, while the majority remained food insecure. Binary logit regression analysis revealed nine significant determinants of household food security, including age, family size, educational level, livestock ownership, and rainfall variability (*p* < 0.05 and *p* < 0.1). Moreover, 82.1% of households adopted climate adaptation strategies, such as soil and water conservation, modified planting time, and improved crop management practices. This result points out the critical need to strengthen household‐level adaptation strategies and improve access to climate information to improve food security in drought‐prone rural areas of Ethiopia.

## 1. Introduction

Climate change has become one of the most significant challenges affecting rural livelihoods and food systems worldwide, particularly in regions reliant on rain‐fed agriculture that are sensitive to climate variations [1]. In addition to gradual changes in temperature and precipitation, climate change increasingly manifests itself through greater climatic variability and extreme weather events, which impact household decision‐making, stability of livelihoods, and food security outcomes [2, 3]. For smallholder farmers in developing nations, these changes result in growing uncertainty regarding agricultural production, income generation, and access to sufficient food [4].

Agriculture is particularly susceptible to climate variability due to its dependence on predictable weather patterns and stable natural resources. A considerable amount of research indicates that climate change disrupts crop growth cycles, diminishes soil moisture and fertility, heightens pest and disease pressures, and worsens water scarcity, all of which collectively threaten agricultural productivity and increase vulnerability in livelihoods [5–7]. However, recent studies underscore that the effects of climate on food security are not solely dictated by exposure [8, 9]. Rather, the outcomes are influenced by the interaction between climatic stress, the sensitivity of livelihoods, household behaviors, and the effectiveness of adaptation strategies. Research conducted by Mosha and Ngulube [10] highlights that adaptation strategies can produce varying results, providing short‐term food access while potentially compromising the long‐term resilience of livelihoods when they depend on depleting assets or reactive coping mechanisms.

Contemporary climate livelihood research is increasingly concentrating on the dynamics at the household level and the behavioral aspects of adaptation [11]. It acknowledges that households exposed to similar climatic conditions often face significantly different vulnerability outcomes. Evidence from Begashaw et al. [12] indicates that climate stress leads to alterations in household production systems, which include modifications in crop‐livestock portfolios and management practices, resulting in uneven effects on food security and the sustainability of livelihoods [13]. These findings highlight the necessity of assessing not only whether households adapt but also the effectiveness of adaptation strategies in mitigating livelihood sensitivity and ensuring food security.

Access to climate information and early warning systems has emerged as a critical factor influencing adaptation behavior [14]. A recent study reveals that households with access to localized climate information are more inclined to implement anticipatory adaptation strategies, make informed decisions regarding planting and resource allocation, and mitigate food‐security risks compared to those who depend on reactive measures [15]. This increasing focus on risk perception, information accessibility, and decision‐making underscores the importance of incorporating behavioral elements into analyses of vulnerability and food security [16, 17].

Concurrent advancements in vulnerability research advocate for a transition toward microscale resilience frameworks that capture the interactions among climate hazards, pathways of livelihood sensitivity, household behavior, and institutional contexts [18]. Research’s in [19] highlights that resilience is developed through localized processes influenced by governance structures, access to knowledge, and the performance of adaptation strategies, rather than relying solely on exposure metrics. Likewise, an additional study emphasizes the significance of modeling household‐level vulnerability to comprehend the reasons behind varying resilience outcomes within the same agro‐ecological context [15].

Ethiopia exemplifies pressing nature of these discussions. Agriculture remains the backbone of the national economy and rural employment; however, it is predominantly reliant on rainfall and is extremely susceptible to climate fluctuations. Unpredictable rainfall patterns, extended dry periods, and increasing temperatures persistently jeopardize food availability, accessibility, and stability, especially in areas prone to drought [8]. Although numerous studies have highlighted the effects of climate on agricultural productivity and food systems at both national and regional levels [20–22], many of these studies regard households as uniform entities or concentrate solely on individual adaptation methods. Recent scholarly works argue that such methodologies fail to account for the sensitivity of livelihoods and the dynamics of land use that influence household vulnerability in the face of climate stress [23–25].

The Enebse Sar Midir District in the Amhara Region represents a highly climate‐exposed yet underresearched context. This district has undergone significant climatic changes in recent decades, marked by erratic rainfall patterns, rising temperatures, and frequent droughts and floods that disrupt agricultural schedules and undermine crop reliability [26]. While some meteorological data indicate slight changes in overall rainfall, the rising intra‐seasonal variability presents considerable challenges for smallholder farmers, exacerbating uncertainty and risks to food security. The growing alternation between extreme wet and dry conditions complicates planting decisions, diminishes crop reliability, and increases vulnerability to food insecurity among smallholder farmers [27]. There is a scarcity of empirical evidence regarding how households in such environments perceive climate risks, choose adaptation strategies, and implement these strategies to enhance food security outcomes.

Recent research in climate change and food security indicates that vulnerability emerges from the interaction of climate hazards, livelihood sensitivity, household behavior, and the effectiveness of adaptation strategies [28–30]. However, many studies in Ethiopia focus primarily on regional or national‐level impacts [31–33], often treating households as homogeneous units and emphasizing the adoption of adaptation practices rather than assessing their effectiveness. Consequently, limited attention has been given to how households at the district level perceive climate risks, manage uncertainty, and implement adaptation strategies to improve food security outcomes.

This research aims to fill these gaps by investigating the vulnerabilities faced by rural households regarding climate change and food insecurity in the Enebse Sar Midir District, utilizing a microscale, household‐centered analytical framework. By employing complementary indicators such as the Household Food Insecurity Access Scale (HFIAS), months of adequate household food provisioning (MAHFP), and the household dietary diversity score (HDDS), the study seeks to capture the multifaceted nature of food security amidst climate stress. Special attention is given to evaluating the effectiveness of household adaptation strategies, which include soil and water conservation practices, modifications in planting schedules, and enhanced agricultural techniques, in mitigating livelihood sensitivity and ensuring food security stability.

This research enhances the comprehension of climate‐livelihood interactions in agricultural systems that are highly vulnerable and reliant on rain by linking climate variability with household perceptions, adaptation behaviors, and the observed outcomes related to food security. The findings contribute to contemporary debates regarding the effectiveness of adaptation strategies, proactive decision‐making, and the development of localized resilience. Furthermore, they offer evidence that can guide the formulation of context‐specific policies and interventions designed to bolster climate resilience and food security in Ethiopia and other drought‐affected areas of sub‐Saharan Africa.

## 2. Conceptual Framework

The research is grounded in the vulnerability–adaptation–resilience (VAR) framework, which offers a comprehensive perspective for analyzing the interactions between climate‐related stresses and household characteristics, along with adaptive responses that affect food‐security outcomes. In this framework, climate exposure refers to the degree to which households encounter climate variability and extremes, including alterations in temperature, fluctuations in rainfall patterns, and the occurrence of extreme events such as droughts and floods, all of which directly influence agricultural production and rural livelihoods [34].

**FIGURE 1:**
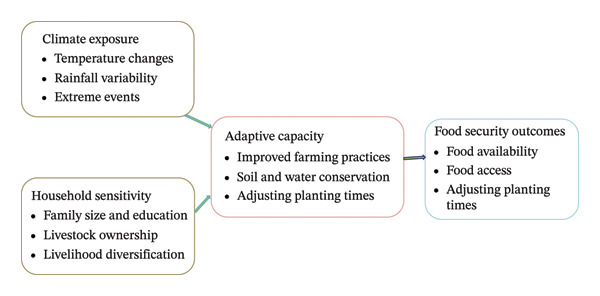
Conceptual framework linking climate exposure, household sensitivity, and adaptive capacity to food security results within the vulnerability–adaptation–resilience (VAR) framework. Adapted from [34, 35].

Household sensitivity indicates the internal socioeconomic and demographic factors that determine how exposure translates into negative impacts. Elements such as family size, educational attainment, livestock ownership, and the degree of livelihood diversification affect households’ dependence on climate‐sensitive resources and their capacity to withstand shocks (Figure 1). Adaptive capacity, a crucial component of the VAR framework, signifies the ability of households to anticipate, manage, and adjust to climate stresses by employing farm‐level adaptation strategies, which encompass soil and water conservation practices, modifications in planting schedules, and enhanced crop and land management techniques, as stated by Kandel et al. [35].

By linking exposure and sensitivity with adaptive capacity, the VAR framework facilitates the examination of how household‐level adaptation alleviates the impacts of climate variability on food security, thereby establishing a robust basis for identifying targeted interventions designed to enhance resilience and diminish vulnerability in rural areas.

## 3. Materials and Methods

### 3.1. Description of the Study Area

**FIGURE 2:**
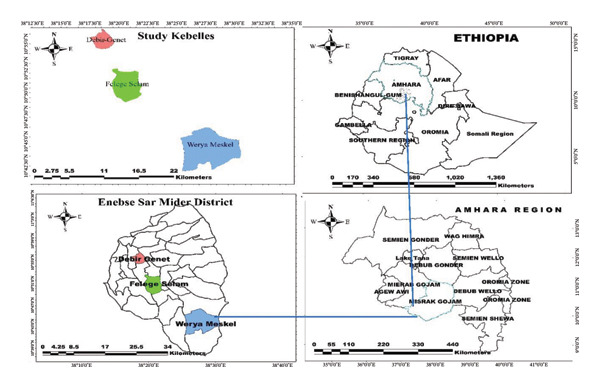
Map of the study area (developed from ArcGIS 10.8 software).

This research was conducted in the Enebsie Sar Midir District, located in the East Gojjam Zone of the Amhara Region in Ethiopia. The district lies between latitudes 10°45′–11°01′ N and longitudes 38°14′–38°18′ E (see Figure 2), with its administrative center, Mertule Mariam, situated approximately 370 km northwest of Addis Ababa, as reported by the Enebsie Sar Midir District Forest and Environment Office (2020). Enebsie Sar Midir encompasses three agro‐climatic zones: Dega (above 2500 m asl), Woyna Dega (1500–2500 m asl), and Kolla (below 1500 m asl). The region experiences a monomodal rainfall pattern, with an average annual precipitation ranging from 900 to 1200 mm, peaking during the rainy season from June to September, as noted by the Enebsie Sar Midir District Forest and Environment Office (2020).

The study district has a total population of 167,347, as reported by the Enebsie Sar Midir Disaster Prevention and Emergency Unit (2020). The district is confronted with ongoing food security challenges, with around 24.4% of its population (40,879 individuals) identified as chronically food insecure and registered as participants in the Productive Safety Net Program (PSNP) since 2005. This significant percentage of PSNP participants reflects a persistent vulnerability to food shortages and a lack of household resilience to various shocks.

The local economy primarily relies on mixed farming systems that integrate crop cultivation and livestock husbandry. Although this approach aims to improve household food availability and diversify income sources, agricultural productivity is hindered by numerous environmental and climatic challenges. As noted by the Enebsie Sar Midir District Forest and Environment Office (2020), the district often experiences irregular and unpredictable rainfall, recurrent droughts, soil erosion, and land degradation, all of which severely impact crop yields and livestock productivity. These obstacles exacerbate chronic food insecurity, especially among resource‐limited households that depend predominantly on rain‐fed agriculture for their livelihoods.

The irrigation infrastructure within the district is inadequately developed, with only 131.5 ha of land currently receiving irrigation through small‐scale schemes. Consequently, agricultural output is significantly reliant on seasonal rainfall, rendering households susceptible to climate variability and production failures, as it was mentioned in the report of the Enebsie Sar Midir District Forest and Environment Office (2020). The restricted access to irrigation, in conjunction with land degradation and persistent drought, limits the district’s ability to attain stable food production and self‐sufficiency.

In general, the district represents a food‐insecure area characterized by chronic dependence on social protection programs, climate‐sensitive livelihoods, and insufficient agricultural infrastructure. These conditions make it an appropriate setting for studying food security dynamics and associated interventions aimed at enhancing household resilience and promoting sustainable livelihoods.

### 3.2. Research Design

A cross‐sectional survey research design was utilized in this study. This design enabled the collection of quantitative data along with some qualitative insights at a specific moment to explore the relationships between food security and climate change‐related factors [36]. The survey methodology facilitated systematic data collection from household participants and facilitated the statistical analysis of essential variables and trends pertinent to the study’s objectives.

### 3.3. Data Type and Sources

The research employed a combination of primary and secondary data sources. Primary data were gathered through a structured household survey administered to sampled households. The survey instrument featured both closed‐ended and open‐ended questions designed to collect quantitative data as well as respondents’ insights regarding food security conditions and the effects of climate change.

Secondary data concerning long‐term temperature and rainfall patterns over the last 3 decades were obtained from the Mertule Mariam weather station and the Regional Meteorology Service Agency (RMSA) located in Bahir Dar. These secondary datasets provided objective climatic information that enhanced the household survey data and facilitated the examination of climate variability and trends within the study region.

### 3.4. Sampling Techniques and Sample Size

The sampling was conducted using a three‐stage design: (1) purposive selection of the Enebse Sar Midir District due to its challenges related to food insecurity; (2) simple random selection of three kebeles (with stratification by agro‐ecological zone [highland, midland, lowland]) from various agro‐ecological zones to ensure a representation of diverse climate and agricultural practices; and (3) systematic random sampling within the three kebeles (small administrative units), with households stratified according to agro‐ecological zone (Table 1). A total sample size of 184 households was determined using the standard sample size formula [37], assuming a margin of error of 7%, which represents a balance between statistical precision and the practical constraints of field‐based data collection in rural settings.

(1) n=N1+Ne2=188811888+0.072=184,

where *N* represents the total population, *n* denotes the sample size, and *e* signifies the acceptable error term.

**TABLE 1:** The distribution of sample households across the selected kebeles.

Kebele	Total HH heads	Sample HHs (M)	Sample HHs (F)	Total sample HHs
Debir Genet	428	36	6	42
Felege Selam	736	56	16	72
Worya Meskel	724	62	8	70
Total	1888	154	30	184

### 3.5. Assessment of Food Security

This research evaluated food security by utilizing key indicators derived from a well‐established framework [38–40] that includes four dimensions: availability, accessibility, stability, and utilization. The main indicators consisted of MAHFP, the HFIAS, and the HDDS. The MAHFP assessed availability by determining the number of months in the past year that households reported having enough food to meet their dietary needs for at least 1 year, taking into account both locally sourced and purchased food items. Households were then classified into three categories of food insecurity: 10–12 months (least insecure), 7–9 months (moderately insecure), and 0–6 months (most insecure), as described by Nanama and Souli [41].

The HFIAS evaluates food access based on subjective responses concerning food scarcity, which includes questions about food shortages, quantity, and the quality of the diet (range: 0–27, with higher scores signifying increased food insecurity) [38]. The HDDS measures food utilization by tallying the number of different food groups consumed in the last 24 h (range: 0–12; where lower scores suggest higher food insecurity). In alignment with previous research [42, 43], the HDDS was categorized as follows: ≤ 5 (indicating low diversity/food insecurity), 6–7 (indicating medium diversity/moderate food insecurity), and ≥ 8 (indicating high diversity/food security). In conclusion, MAHFP and HFIAS assessed food availability and access, whereas HDDS evaluated dietary quality and utilization. Furthermore, the findings from the other three components were examined to explore the stability dimension, which encompasses all these factors [44, 45].

### 3.6. Data Analysis Method

Data analysis was conducted using Microsoft Excel and SPSS Version 26. The data were examined through descriptive statistics (which included means, frequencies, and cross‐tabulation), inferential statistics (such as chi‐squared tests, *p*‐values, and *t*‐tests), and binary logit regression to determine the factors affecting household food security. The dependent variable, HDDS, was recoded to reflect food secure (HDDS ≥ 8) or food insecure (HDDS < 8).

The independent variables comprised gender, age, education, family size, off‐farm participation, farmland size, access to irrigation, income, frequency of droughts/floods, and access to weather forecasts, agricultural extension services, credit, livestock ownership, market distance, and climate change. In accordance with the methodology described in references [46, 47], the binary logistic regression model was constructed as follows:

(2) Pi=PY=1Xi=11+e−βo+βjXi,

where *Pi* represents the probability that the household is food‐secure. *X* *i* denotes the set of explanatory variables for the *i*th household. *β* *o* and *β* *j* are the parameters to be estimated.

By substituting *β* *o* + *β* *j* *X* *i* by *Z* *i*,

(3) Pi=11+e−Zi=eZi1+eZi,

where *Pi *= (*Y* = 1) signifies the probability of food insecurity

(4) 1−Pi=11+eZi.

Odds ratio for food security—the odds ratio favoring food security is expressed as

(5) Pi1−Pi=eZi/1+eZi1/1+eZi=eZi.

The logit model transformation, which presumes a linear relationship between the outcome variable and the explanatory variables, is represented as

(6) Ln Pi1−Pi=Zi=β0+βjXi+ɛi,

where *β*0 is the constant, and *β* *i*, where *i* = 1, 2,…, *j*, are the coefficients of the explanatory variables to be estimated, *ɛ* *i* is represents the error term for the *i*th observation.

## 4. Results and Discussion

### 4.1. Household Demographic and Socioeconomic Characteristics

Table 2 presents the demographic and socioeconomic attributes of the sampled households categorized by their food security status. The average age of household heads was 47.9 years (SD = 10.3). Households that were food‐secure were led by significantly older individuals (mean = 53.24 years) in comparison to those that were food‐insecure (mean = 45.35 years), with this difference being statistically significant (*t* = 5.19, *p* < 0.01). This observation implies that older household heads may gain advantages from accumulated farming experience, enhanced social networks, and greater asset holdings, which improve their ability to manage climatic risks and maintain food availability. Comparable evidence from pastoral and agrarian systems suggests that knowledge derived from experience aids in adaptive decision‐making during climate stress, especially in contexts where formal safety nets are lacking [48]. However, age may also exert a dual influence, as younger farmers, despite having less experience, often possess higher levels of formal education and may be more open to adopting climate‐smart innovations, indicating that adaptive capacity is influenced by both experiential and human capital factors.

**TABLE 2:** Household demographic, socioeconomic characteristics, and perceptions of climate change by food security status.

Variable	Category/unit	Food‐secure (*N* = 59)	Food‐insecure (*N* = 125)	Total (*N* = 184)	Statistical test
*Continuous variables (mean ± SD)*					
Age of HH head	Years	53.24 ± 9.70	45.35 ± 9.58	184	*t* = 5.19^∗∗∗^

Family size	Number	6.17 ± 1.40	6.23 ± 1.45	184	*t* = −0.28

Landholding size	ha	1.00 ± 0.36	0.41 ± 0.19	184	*t* = 14.74^∗∗∗^

Livestock ownership	TLU	8.00 ± 3.60	2.16 ± 1.39	184	*t* = 15.86^∗∗∗^

*Categorical variables (no. and %)*					
Sex of HH head	Male	55 (93.2%)	99 (79.2%)	154 (83.7%)	χ^2^ = 5.77^∗∗^
	Female	4 (6.8%)	26 (20.8%)	30 (16.3%)	

Marital status	Married	55 (93.2%)	102 (81.6%)	157 (85.3%)	χ^2^ = 10.07^∗∗∗^
	Divorced	1 (1.7%)	21 (16.8%)	22 (12.0%)	
	Widowed	3 (5.1%)	2 (1.6%)	5 (2.7%)	

Education level	Literate	53 (89.8%)	12 (9.6%)	65 (35.3%)	χ^2^ = 112.93^∗∗∗^
	Illiterate	6 (10.2%)	113 (90.4%)	119 (64.7%)	

*Climate change perceptions*					
Perceived change in rainfall	Yes	—	—	166 (90.2%)	—
	No	—	—	18 (9.8%)	—

Perceived change in temperature	Yes	—	—	158 (85.9%)	—
	No	—	—	26 (14.1%)	—

*Note:* ha = hectare; *t* = *t*‐test; χ^2^ = chi‐square test.

Abbreviations: SD, standard deviation; TLU, tropical livestock unit.

^∗∗^ *p* < 0.05.

^∗∗∗^ *p* < 0.01.

The size of households did not reveal a statistically significant difference between food‐secure and food‐insecure households (6.17 vs. 6.23 members; *t* = −0.28, *p* = 0.783). This indicates that family size by itself was not a critical determinant of food security in the area studied. The findings suggest that labor availability is not a significant limitation to agricultural production or adaptation, as the majority of households depend on family labor for routine farming activities and climate‐related practices such as soil and water conservation and land management. Similar findings in rural livelihood systems emphasize that labor endowment only leads to food security when it is supported by productive assets and institutional support [49].

In contrast, the size of landholdings and ownership of livestock showed strong and highly significant differences between food‐secure and food‐insecure households. Households that are food secure possessed considerably larger landholdings (mean = 1.00 ha) compared to those that are food insecure (mean = 0.41 ha; *t* = 14.74, *p* < 0.01). A larger land area enhances production capacity, promotes crop diversification, and facilitates investment in soil and water conservation practices, thereby mitigating vulnerability to fluctuations in rainfall. Similarly, livestock ownership was significantly greater among food‐secure households, averaging 8.00 tropical livestock units (TLUs), in contrast to just 2.16 TLU for food‐insecure households (*t* = 15.86, *p* < 0.01) (Table 2). Livestock fulfill various roles as sources of food, income, manure, and financial safety nets during climatic disturbances and thus play a central role in strengthening household resilience. This observation aligns with recent findings [50] indicating that effective livestock management systems considerably lower mortality risks and improve household income and food security amidst changing climatic conditions.

With respect to categorical variables, male‐headed households represented 83.7% of the sample and were significantly more likely to achieve food security compared to female‐headed households (*χ* ^2^ = 5.77, *p* < 0.05). This difference highlights structural gender inequalities in access to land, livestock, credit, extension services, and climate information. Similar studies in developing regions underscore that gender‐specific access to institutional services limits women’s adaptive capacity and resilience in their livelihoods in the face of climate change [49]. Additionally, marital status exhibited a significant association with food security (*χ* ^2^ = 10.07, *p* < 0.01), with married households being more food secure than those that are divorced or widowed. Married households may gain advantages from shared labor, pooled resources, and more robust social support networks, which are essential for managing climate‐induced shocks.

Education emerged as a significant factor influencing food security. Nearly 90% of household heads who are food‐secure possess literacy skills, in contrast to over 90% of food‐insecure household heads who are illiterate (*χ* ^2^ = 112.93, *p* < 0.01). Literacy plays a crucial role in enhancing access to information regarding climate and markets, fostering engagement with extension services, and promoting the adoption of advanced agricultural technologies. Empirical evidence from studies [48] on climate adaptation and rural development consistently underscores the importance of education as a fundamental element of adaptive capacity and sustainable food security.

Moreover, perceptions regarding climate change were widespread across the sample, with 90.2% of households reporting changes in rainfall patterns and 85.9% noting increases in temperature. Although these perceptions did not differ based on food security status, they reinforce the conclusive notion that climate change is a tangible reality within the study area. This collective awareness serves as a vital foundation for enhancing collaborative adaptation strategies, extension initiatives, and climate‐smart interventions, as highlighted in the wider literature on climate resilience [51].

### 4.2. Trends in Rainfall and Temperature Analysis Using Meteorological Data

**FIGURE A1:**
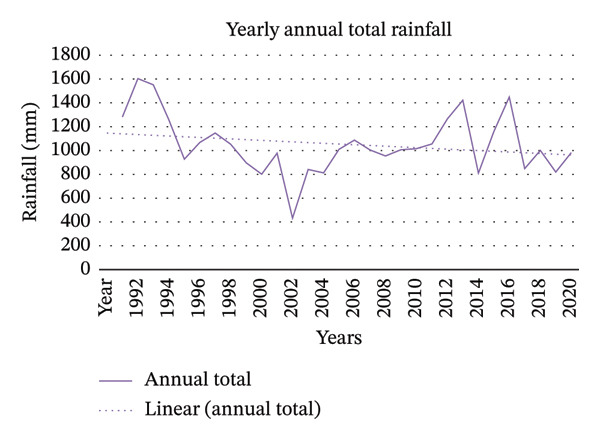
Annual mean rainfall trends (1991–2020). *Source:* NMA, from Mertule Mariam station.

**FIGURE A2:**
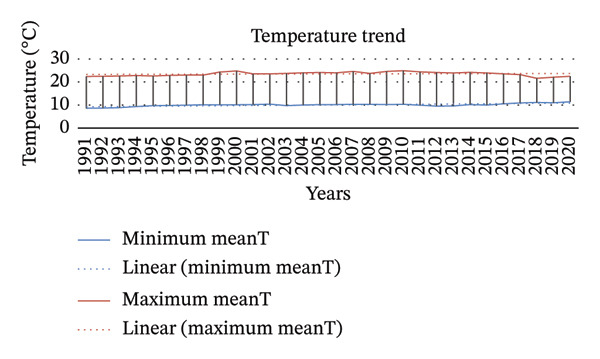
Annual mean (maximum and minimum) temperature trend (1991–2020). *Source:* NMA, from Mertule Mariam station.

An analysis of meteorological data from the Mertule Mariam weather station for the period 1991–2020 indicates a significant decrease and heightened variability in annual rainfall within the Enebse Sar Midir District (Appendix B Table A4; Appendix B Figures A1 and A2). The average annual rainfall during this timeframe was approximately 1051.4 mm; however, the long‐term trend suggests a gradual decline in total annual rainfall of about 6.11 mm per year, alongside an increase in interannual variability. Certain years experienced notably high rainfall, such as 1992 with 1603.7 mm, while others recorded alarmingly low totals, including 2002 (432.1 mm) and 2014 (810.1 mm). These variations illustrate a diminishing reliability of the rainfall patterns that support rain‐fed agriculture in the district.

The noted decline in rainfall aligns with previous research conducted in Ethiopia. For example, Gessese [52] identified decreasing rainfall trends in Debre Berhan and Ziway through long‐term meteorological data, while Osman [53] observed similar reductions in the central highlands. In addition to the overall decrease in rainfall, the distribution of precipitation has become more erratic, characterized by unpredictability in the onset, duration, and conclusion of the rainy season. This instability disrupts traditional agricultural schedules, heightening the risk of delayed planting, crop failures, and yield reductions, especially in years marked by extended dry periods or premature cessation of rains.

Extreme rainfall events exacerbate these issues. Years characterized by above‐average precipitation, such as 1992 and 2016, stand in stark contrast to drought years like 2002, 2014, and 2019. The fluctuations between droughts and heavy rainfall events increase vulnerability to both moisture stress and flooding. Short‐lived, high‐intensity rainfall occurrences have led to soil erosion, waterlogging, and land degradation, which in turn threaten agricultural productivity and the long‐term sustainability of land. These trends correspond with broader climate change indicators noted throughout sub‐Saharan Africa, where variability in rainfall has emerged as a significant source of risk to livelihoods.

Participants consistently pointed out the severe drought periods of 2002/2003 and 2014/2015 as times marked by critical food shortages, widespread crop failures, and a heightened dependence on external food aid. The unpredictability of rainfall has particularly impacted staple crops such as teff, sorghum, wheat, and beans, especially during vital growth phases in the primary cropping season. These observations align with Tofu et al. [23], which identified the unpredictability of rainfall as a key factor contributing to agricultural vulnerability and food insecurity in Ethiopia.

An analysis of temperature trends further reveals a warming pattern in the study region. Data from the National Meteorological Agency indicate that maximum temperatures in Enebse Sar Midir have risen by an average of 0.14°C per decade during the study period, surpassing the national average increase of approximately 0.10°C per decade. Minimum temperatures have also shown an upward trend, increasing by about 0.0°C per decade, which aligns with national data reported by Fazzini et al. [54]. These trends are consistent with household perceptions, as a significant majority of respondents noted observable increases in temperature (Appendix B Table A4; Appendix B Figures A1 and A2).

Rising temperatures, particularly the increase in minimum temperatures, have significant implications for agricultural systems and the livelihoods of rural communities. Higher temperatures accelerate evapotranspiration, intensify moisture stress, and increase the water requirements for crops, thereby worsening the negative impacts of decreasing and unpredictable rainfall. Evidence from rural systems affected by climate change suggests that rising temperature extremes considerably weaken adaptive capacity, particularly in areas where access to irrigation, extension services, and institutional support is limited [48].

The strong correlation between household perceptions and recorded meteorological trends emphasizes that climate change is not merely a scientific issue but also a tangible experience in the study area. This alignment underscores the necessity of reinforcing localized adaptation strategies and institutional responses. Research from other African regions indicates that focused institutional services, such as the dissemination of climate information, the development of water management infrastructure, and the provision of extension support, are vital in bolstering household resilience amid increasing climatic uncertainty [51]. In the absence of such interventions, ongoing warming and rainfall variability are expected to exacerbate food insecurity and undermine the sustainability of rain‐fed livelihoods in the Enebse Sar Midir District.

### 4.3. Perceptions of Climate Change

#### 4.3.1. Perceived Trends in Temperature and Rainfall

Households’ perceptions on climate change, as summarized in Figure 3, reveal a significant level of awareness regarding long‐term climatic alterations in the study region. A considerable majority of participants (85.9%) reported observable temperature changes over the last 30 years, while an even larger segment (90.2%) recognized substantial shifts in rainfall patterns. These results imply that climate change is broadly acknowledged at the household level and is increasingly perceived as a persistent challenge impacting agricultural productivity and rural livelihoods, as discussed by Dube and Phiri [55].

FIGURE 3Respondents’ perception regarding long‐term climate exposure, specifically temperature and rainfall trends, over the last 3 decades. Note: The letter (a) indicates perceived trends in temperature, whereas the letter (b) depicts perceived changes in rainfall patterns.

**Figure:**
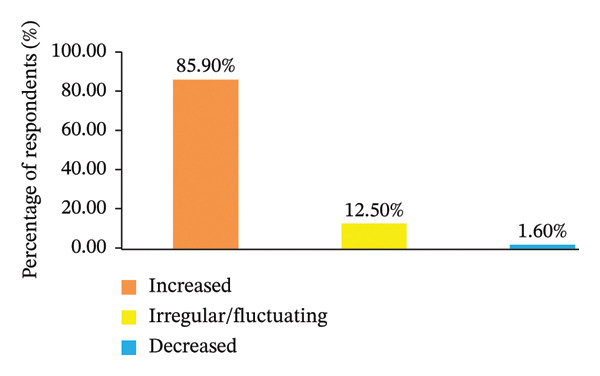
(a)

**Figure:**
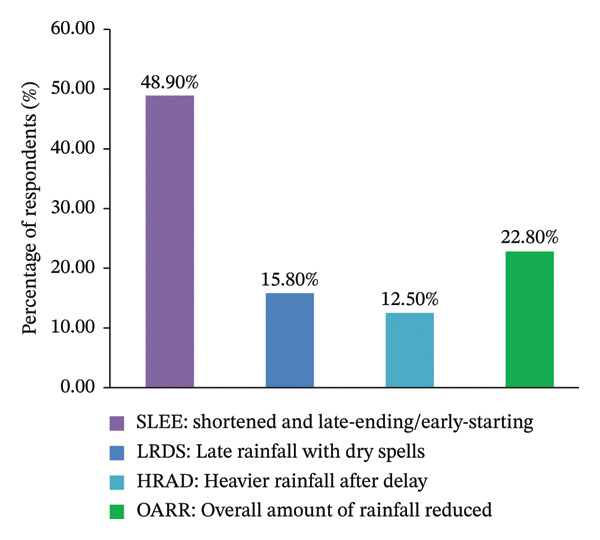
(b)

This extensive awareness is particularly crucial in a primarily rain‐fed agricultural system, where variations in temperature and precipitation directly affect planting choices, crop yields, and food security. The perception of climate change is vital in influencing household responses, as it dictates whether farmers will pursue proactive adaptation strategies or depend on reactive coping mechanisms. Recent research highlights that households with increased climate awareness are more inclined to implement adaptive measures, assuming that there is adequate institutional support and access to information [56].

Concerning prolonged exposure to temperature, a significant majority of respondents (85.9%) perceived a general rise in temperature over the last 3 decades, whereas 12.5% characterized temperature trends as increasingly erratic or variable (Figure 3(a)). Only a very small proportion (1.6%) indicated a perceived reduction in temperature. These perceptions are indicative of farmers’ direct experiences with escalating heat stress, reduced growing seasons, and heightened evapotranspiration, aligning with the documented meteorological patterns in the region. Comparable findings from climate‐affected rural areas suggest that increasing temperatures exacerbate livelihood vulnerability by elevating production risks and diminishing adaptive capacity, especially in contexts where access to climate‐resilient technologies is constrained [57].

Furthermore, perceptions regarding rainfall patterns underscore the rising uncertainty and instability (Figure 3(b)). Almost half of the participants (48.9%) reported that the rainy season now commences later and concludes earlier than previously observed, while 15.8% reported delayed rainfall accompanied by extended dry periods. Additionally, 12.5% witnessed a combination of delayed onset and more intense rainfall occurrences, and 22.8% perceived an overall reduction in total rainfall. Collectively, these observations indicate a disruption of traditional seasonal cycles, complicating agricultural planning, crop selection, and labor distribution in a farming system that remains significantly reliant on rainfall.

The variability in perceived rainfall has significant consequences for food security, as both delayed onset and premature cessation of rains heighten the risk of crop failure and diminish yield stability. Research from various African dry‐land and rain‐fed systems suggests that the unpredictability of rainfall compels households to implement short‐term coping mechanisms, which may provide temporary stabilization of food access but can weaken long‐term resilience if not supported by effective institutional services [51]. In this regard, perception alone is insufficient; the conversion of climate awareness into successful adaptation is largely contingent upon access to extension services, climate information, and coordinated institutional support [58].

The alignment between household perceptions and meteorological data enhances the credibility of local knowledge as a vital component in climate adaptation planning. Comparable observations have been documented throughout Ethiopia and other regions of Africa, where farmers commonly recognize trends of increasing temperatures, delayed rainfall onset, and shortened rainy seasons that closely correspond with instrumental climate records [59–61]. This alignment highlights the necessity of merging farmers’ experiential knowledge with scientific climate data to formulate locally relevant, socially acceptable, and effective adaptation strategies.

Overall, the high level of climate awareness observed in Enebse Sar Midir provides a strong foundation for resilience‐building initiatives. Nevertheless, in the absence of supplementary investments in institutional services, climate information systems, and adaptive capacity, an increased perception of climate risk may lead to increased anxiety rather than effective adaptation. Therefore, it is crucial to strengthen the connection between climate perception, informed decision‐making, and practical adaptation support to enhance household food security amidst growing climatic uncertainty.

#### 4.3.2. Perceived Consequences of Climate Change on Household Food Security

Climate change is regarded as a significant and growing threat to household food security within the study region, where the livelihoods of the population predominantly rely on rain‐fed agriculture, making them particularly vulnerable to climatic fluctuations. As shown in Table 3, a substantial majority of households (79.3%) have observed a marked increase in the frequency of droughts over the last 20–30 years. The perceptions regarding the occurrence of droughts varied among different kebeles, with reports ranging from 73.6% in Felege Selam to 88.6% in Worya Meskel. The Pearson chi‐square test revealed a marginally significant difference across kebeles (*χ* ^2^ = 5.866, df = 2, *p* = 0.053), indicating spatial variability in climate exposure, which may be associated with microclimatic conditions, topographical features, and unequal access to adaptive resources.

**TABLE 3:** Perceived changes in drought and flood occurrence across kebeles over the past 20–30 years.

Kebele	Drought occurrence yes (no. %)	Drought occurrence no (no. %)	Flood occurrence yes (no. %)	Flood occurrence no (no. %)	Total (no. %)
Felege Selam	53 (73.6%)	19 (26.4%)	46 (63.9%)	26 (36.1%)	72 (100%)
Worya Meskel	62 (88.6%)	8 (11.4%)	42 (60.0%)	28 (40.0%)	70 (100%)
Debir Genet	31 (73.8%)	11 (26.2%)	37 (88.1%)	5 (11.9%)	42 (100%)
Total	146 (79.3%)	38 (20.7%)	125 (68.0%)	59 (32.0%)	184 (100%)

*Note:* Pearson chi‐square for drought occurrence: *χ* ^2^ = 5.866, df = 2, *p* = 0.053; percentages are calculated within kebeles.

Drought has been consistently recognized as the most critical climate‐related threat to household food security. Respondents highlighted that extended dry periods and the late onset of rainfall often result in moisture stress during essential crop growth phases, leading to either partial or complete crop failure. Similar patterns have been observed in other rural systems vulnerable to climate change, where production shocks caused by drought diminish food availability, weaken household income, and compel reliance on detrimental coping mechanisms, as discussed by Mtetwa [62]. In these scenarios, repeated exposure to drought not only impacts immediate food access but also jeopardizes long‐term livelihood resilience by exhausting productive resources.

**FIGURE 4:**
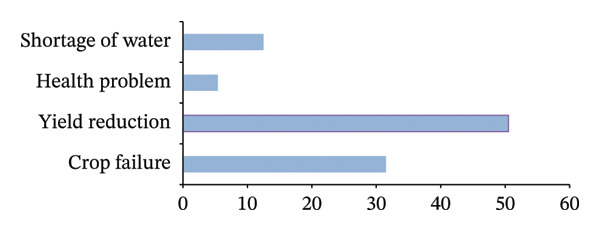
Problems faced due to drought.

In addition to drought, flooding has emerged as a significant and compounding hazard. In total, 68.0% of households indicated they had encountered floods during the same timeframe, with an especially high occurrence in Debir Genet (88.1%). Flood events were frequently linked to soil erosion, damage to crops, loss of stored food, and the destruction of household assets (Figure 4). Although flooding is less common than drought, its effects are often abrupt and severe, exacerbating land degradation and diminishing future agricultural productivity [63]. The simultaneous occurrence of both droughts and floods reflects the increasing variability of climate and the rising unpredictability of extreme events impacting local agricultural systems.

The simultaneous impacts of drought and flooding significantly increase household vulnerability by disrupting food availability, access, and stability. Decreased crop yields lead directly to diminish household incomes, food shortages, and escalating food prices, especially in rain‐fed systems where production margins are already limited. Empirical research from Ethiopia demonstrates that even minor climatic disturbances can result in disproportionately severe effects on food security in these circumstances [64–66]. Moreover, rising temperatures further worsen yield reductions by enhancing evapotranspiration and increasing crop water needs, aligning with studies indicating that temperature rises considerably diminish crop productivity in rain‐fed agricultural systems [67].

Figure 4 depicts the key challenges encountered by households as a result of drought, which encompass diminished crop yields, total crop failure, scarcity of water, and increased health‐related issues. These effects interact in ways that exacerbate food insecurity, especially for households with limited resources that lack access to irrigation, credit, or alternative income sources. Evidence from rural areas affected by climate stress indicates that, in the absence of sufficient institutional support, households tend to rely on short‐term coping strategies that may temporarily stabilize food access but ultimately undermine long‐term adaptive capacity [68].

**FIGURE 5:**
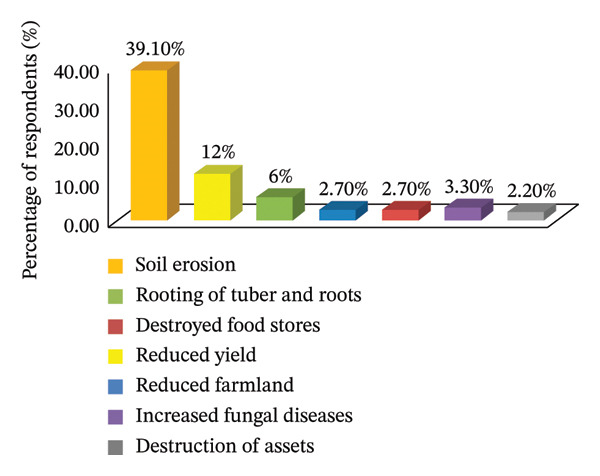
Challenges related to flooding encountered by respondents.

Participants also reported significant changes in local weather patterns, particularly regarding the timing and intensity of rainfall. Historically, rainfall was fairly consistent from April to September; however, it has now shifted to occur in shorter, more intense, and unpredictable incidents. Such changes in rainfall patterns lead to inadequate soil moisture absorption, increased runoff, and drought‐like conditions, even in the presence of occasional heavy rains. These changes have resulted in a decline in both groundwater and surface water availability, a rise in pest and disease outbreaks, and further decreases in crop yields (Figure 5). These observations align with meteorological data from the Mertule Mariam weather station (1991–2020), thereby reinforcing the validity of local experiential knowledge.

In general, the observed rise in climate extremes, especially droughts and floods, emphasizes the significant vulnerability of rural livelihoods in the Enebse Sar Midir District. The findings highlight the pressing necessity for integrated adaptation strategies that address various, interconnected climate risks instead of focusing on single hazards in isolation. Evidence from other regions in Africa indicates that strengthening institutional services, enhancing water management infrastructure, and improving access to climate information are essential for mitigating climate‐induced food insecurity and building long‐term resilience [51]. In the absence of such measures, ongoing climate variability is expected to further jeopardize food security and the sustainability of livelihoods in the area under study.

### 4.4. Food Security Analysis of the Household

#### 4.4.1. Food Availability

The availability of food within households was evaluated through the MAHFP indicator, which measures the number of months in a year that households can fulfill their food requirements from their own production and other sources. The findings reveal a significant level of food insecurity in the examined region. In total, 66.3% of households were identified as food insecure, having adequate food provision for less than 10 months each year, whereas only 33.7% of households were deemed food secure, possessing enough food for a minimum of 10 months per year (Table 4).

**TABLE 4:** Number of months respondents consume their own products.

Kebele	Most food‐insecure		Moderately food insecure	Food‐secure	Total
	1–3 months	4–6 months	7–9 months	10–12 months	
Debir Genet	0	10 (23.8%)	15 (35.7%)	17 (40.5%)	42 (100%)
Felege Selam	1 (1.4%)	7 (9.7%)	35 (48.6%)	29 (40.3%)	72 (100%)
Worya Meskel	6 (8.6%)	29 (41.4%)	19 (27.1%)	16 (22.9%)	70 (100%)
Total	7 (3.8%)	46 (25%)	69 (37.5%)	62 (33.7%)	184 (100%)

*Note:* Pearson chi^2^ = 29.534, *p* < 0.001.

Food availability exhibited considerable variation across kebeles (*χ* ^2^ = 29.534, *p* < 0.01), underscoring spatial disparities in vulnerability. Worya Meskel demonstrated the highest levels of food insecurity, with nearly half of the households categorized as being in the most food‐insecure brackets (1–6 months of food self‐sufficiency). In contrast, Debir Genet and Felege Selam displayed relatively improved food availability, with around 40% of households deemed food secure. These discrepancies may be indicative of differences in agro‐ecological conditions, land productivity, asset ownership, and access to adaptive resources such as livestock, labor, and soil and water conservation practices.

Throughout all kebeles, the majority of households were found in the “moderately food insecure” category (7–9 months of food availability), suggesting that while most households can satisfy their food requirements for part of the year, they remain significantly vulnerable to seasonal shortages and climatic disturbances. This trend implies that food availability in the study region is fragile and highly responsive to fluctuations in rainfall, crop yields, and access to agricultural inputs. Similar findings have been documented in climate‐sensitive rural systems, where households frequently achieve temporary food sufficiency but lack the resilience necessary to maintain an adequate food supply year‐round [69].

Respondents consistently reported that food shortages become more pronounced following Ethiopian Easter, with the most critical period occurring between June and September. During this interval, household food reserves are diminished, market prices escalate, and the demand for agricultural labor increases, while crop harvests have yet to begin. This cyclical pattern of seasonal hunger reflects the structural vulnerabilities in food availability and emphasizes the instability aspect of food security.

The recurring seasonal depletion of food supplies suggests that numerous households depend on short‐term coping strategies, including decreasing meal frequency, selling livestock, or borrowing food to fill food gaps. Although these approaches may provide temporary relief in food access, they can weaken long‐term resilience by depleting productive assets and constraining future production capabilities. Evidence from various agricultural settings indicates that households with more robust asset bases and superior management systems, especially those that combine livestock and crop production, are better able to buffer seasonal food shortages and stabilize food availability [50].

Overall, the MAHFP findings reveal that food availability in the Enebse Sar Midir District is not only insufficient for a significant number of households but also highly inconsistent across different seasons. These findings highlight the necessity for targeted interventions aimed at enhancing household production capacity, improving storage and postharvest management, and promoting climate‐resilient agricultural practices. According to Ullah et al. [49], strengthening institutional support, such as extension services, better access to inputs, and climate‐informed production planning, has been demonstrated to be crucial in enhancing food availability and decreasing vulnerability in rural areas affected by climate change. In the absence of such initiatives, seasonal and climate‐related food shortages are likely to continue, further jeopardizing household food security and resilience.

#### 4.4.2. Examination of Food Access Through the Household

Household food access was evaluated using the HFIAS, which measures anxiety regarding food supply, limitations on food quality, and reductions in food quantity over a 1‐month recall period. The findings suggest that food access‐related insecurity is prevalent in the study area. In total, 67.4% of households faced some form of food access constraint during the recall period, indicating significant vulnerability to short‐term shocks and seasonal shortages.

The distribution of responses to the 9 HFIAS occurrence questions (Table 5) reveals that food insecurity manifested through various and overlapping dimensions. A considerable number of households expressed frequent concerns about inadequate food availability, the inability to consume preferred foods, dependence on a limited variety of foods, and the necessity to consume less desirable food items. Constraints related to quantity were also significant, with many households reporting smaller meal sizes, fewer meals per day, and periods when no food was available at home. While complete fasting for an entire day and night was relatively uncommon, the widespread presence of less severe but persistent access constraints suggests chronic food stress rather than isolated shocks.

**TABLE 5:** Distribution of households according to HFIAS condition.

HFIAS condition	Rarely (%)	Sometimes (%)	Often (%)
1. Concerned about insufficient food availability	51.6	25.0	23.4
2. Unable to eat preferred food varieties	32.3	36.3	31.5
3. Limited to a narrow range of food options	30.6	39.5	29.8
4. Require to consume some undesirable food items	32.7	37.9	22.6
5. Consumed smaller portions than necessary	39.5	40.3	30.6
6. Had fewer meals each day	38.5	40.2	21.3
7. No food available at home	33.1	48.3	18.6
8. Experienced hunger due to food scarcity	32.1	46.8	21.1
9. Went without food for the entire day and night	48.8	51.2	0.0
Total score	36.9	40.0	23.1

Severity analysis further underscores the extent of food access insecurity. Based on the frequency of occurrence, 36.9% of households reported experiencing food access issues rarely, 40.0% sometimes, and 23.1% often during the 1‐month recall period. The mean HFIAS score was 12.36 (SD = 8), indicating moderate overall food access insecurity at the population level. Utilizing established cutoff points [70], 42.9% of households were categorized as most food secure (HFIAS ≤ 11), 23.4% as moderately food insecure (scores 12–16), and 33.7% as severely food insecure (scores ≥ 17).

These findings indicate that although some households can sustain food access under typical circumstances, a significant number face ongoing and severe limitation that affect both the quality and quantity of their diet. Such trends are typical of agricultural systems that are rain‐fed and exposed to climate variability, where income instability, seasonal production patterns, and limited market integration increase susceptibility to food access shocks. Evidence from similar rural settings shows that households with diversified income sources, stable revenue sources, and productive assets, particularly livestock, are more capable of mitigating food access challenges during stressful periods [71].

In alignment with the work of Gebreyesus et al. [72], the analysis of HFIAS results is enhanced by examining the categories of household food insecurity access prevalence (HFIAP), which differentiate between food‐secure, mildly food‐insecure, moderately food‐insecure, and severely food‐insecure households based on the severity and frequency of their reported experiences. Within this framework, households reporting more severe indicators such as decreased meal frequency, hunger, or insufficient food at home are categorized into higher severity levels, indicating more profound and persistent challenges in accessing food [73].

From the perspective of vulnerability, adaptation, and resilience, the widespread occurrence of food access insecurity highlights the interaction between climatic stress, sensitivity of livelihoods, and limited capacity to adapt. Without sufficient institutional support, households frequently depend on short‐term coping mechanisms such as borrowing food, selling livestock, or cutting back on consumption, which may provide temporary stabilization of access but can compromise long‐term resilience. Recent studies underscore the importance of enhancing institutional services, providing extension support, and creating income‐generating opportunities as essential for reducing food access vulnerability and empowering households to proactively address climate‐related shocks [74].

Overall, the findings from the HFIAS indicate that food access within the Enebse Sar Midir District is significantly limited and unevenly allocated among households. To effectively tackle food access insecurity, it is essential to implement integrated strategies that extend beyond merely enhancing food availability; these strategies should also encompass income stabilization, improved market access, and the establishment of institutional support systems that can protect households from both climatic and economic shocks.

#### 4.4.3. Food Utilization Analysis

The assessment of food utilization, which pertains to the quality and nutritional sufficiency of diets, was conducted using the HDDS. The HDDS scores for the households surveyed varied from 2 to 9 food groups, with an average score of 5.74, signifying a notable diversity in dietary quality throughout the study region. This variability is indicative of the differences in households’ access to a range of food sources, their income levels, and their capacity to withstand climate‐related challenges. Significant disparities were observed among the three kebeles, particularly with households in Worya Meskel demonstrating the least dietary diversity.

According to the established thresholds for the HDDS, households that score five or lower are classified as food insecure, while those with scores ranging from six to seven are categorized as experiencing moderate food insecurity, and scores of eight or higher denote food security [42, 43]. The findings indicate a disparity in dietary quality and food utilization results. As shown in Table 6, Felege Selam exhibited the highest percentage of households with an HDDS of six (40.3%), which implies a relatively improved access to a variety of foods, although still classified within the moderate food insecurity range. Furthermore, 37.5% of households in this kebele attained an HDDS of eight, signifying food security. In contrast, Worya Meskel reported the lowest dietary diversity, with only 15.7% of households achieving an HDDS of two, indicative of a highly restricted diet characterized by a limited selection of food items. Debir Genet, on the other hand, showed comparatively favorable results, with 38.1% of households reaching an HDDS of eight, reflecting a relatively enhanced food utilization and nutritional security.

**TABLE 6:** Household dietary diversity score of respondents.

HDDS	Debir Genet	Felege Selam	Worya Meskel	Total
2	0	0	11 (15.7%)	11 (6%)
3	2 (4.8%)	0	14 (20%)	16 (8.7%)
4	9 (21.4%)	1 (1.3%)	13 (18.6%)	23 (12.5%)
5	11 (26.2%)	12 (16.7%)	12 (17.1%)	35 (19%)
6	3 (7.1%)	29 (40.3%)	3 (4.3%)	35 (19%)
7	1 (2.4%)	3 (4.2%)	1 (1.4%)	5 (2.7%)
8	16 (38.1%)	27 (37.5%)	14 (20%)	57 (31%)
9	0 (0%)	0 (0%)	2 (2.9%)	2 (1.1%)
Total	42 (100%)	72 (100%)	70 (100%)	184 (100%)

*Note:* HDDS ≤ 5 indicates greater food insecurity, HDDS 6‐7 indicates medium food insecurity, and HDDS ≥ 8 indicates food security.

Low dietary diversity (HDDS ≤ 5) was noted in 46.2% of households, highlighting the extent of food insecurity associated not only with inadequate food quantity but also with poor diet quality. Additionally, 21.7% of households were categorized under medium dietary diversity (HDDS 6‐7), indicating moderate food insecurity, while only 32.1% achieved high dietary diversity (HDDS ≥ 8). These results imply that almost two‐thirds of households do not have reliable access to a sufficiently varied diet to fulfill their nutritional needs.

The disparities observed in dietary diversity are closely connected to livelihood assets and the capacity for income generation. Evidence from various rural and agro‐pastoral settings indicates that having diversified income sources, especially through livestock ownership and enhanced livestock management, significantly improves households’ ability to obtain a range of nutritious foods [75]. Livestock not only supply animal‐source foods but also act as a crucial income safeguard, allowing households to buy diverse food products during times of climatic distress. This observation corresponds with the relatively elevated HDDS found in kebeles where livestock ownership and income opportunities are more evident.

Furthermore, climate variability exacerbates challenges related to food utilization by disrupting food production and restricting market access. Frequent droughts and unpredictable rainfall diminish crop yields and limit households’ ability to diversify their diets, particularly in regions like Worya Meskel. Similar trends have been observed in climate‐sensitive areas of Africa, where limited adaptive capacity and inadequate institutional support compromise dietary quality, even though households are aware of climate‐related risks [76]. Access to institutional services, such as extension support and climate adaptation initiatives, has been demonstrated to be vital in strengthening household resilience and enhancing food and nutrition outcomes.

Furthermore, interventions based on landscape, including agroforestry, afforestation, and the restoration of forest landscapes, can have an indirect impact on food utilization by improving ecosystem services, stabilizing livelihoods, and supporting diversified production systems. Studies conducted in similar contexts indicate that community‐driven agroforestry and restoration projects enhance livelihood resilience and food accessibility when they are inclusive and aligned with local requirements [77]. However, restrictive land‐use interventions without adequate livelihood alternatives may negatively affect food access and dietary diversity, especially for households with limited resources [78].

In conclusion, the findings indicate that food utilization in the examined area is limited by a combination of climatic challenges, restricted income opportunities, and unequal access to productive resources and institutional support. To enhance dietary diversity, particularly in highly vulnerable kebeles like Worya Meskel, it is essential to implement integrated interventions that strengthen climate resilience, increase livestock and crop productivity, promote diversified livelihoods, and ensure that adaptation and natural resource management initiatives support, rather than undermine household food and nutrition security.

#### 4.4.4. Food Stability in the Study Area

Food stability, which refers to the consistent ability of households to access adequate food over time, is a fundamental component of food security. In the study area, food stability has been significantly compromised by recurrent droughts and other extreme climate events, which disrupt agricultural production, diminish household incomes, and limit market access. Data from the Food Security Process Unit of the Enebse Sar Midir District Office of Agriculture reveals a gradual decline in food stability due to the occurrence of repeated droughts in recent years.

Severe food shortages were documented in 2014, 2015, and 2019, each coinciding with harsh drought conditions. In 2014, around 296,910 households were impacted, while 108,018 households faced food insecurity in 2015 and 46,068 households in 2019. The drought of 2015 was especially severe, affecting 24 out of the 33 rural kebeles and necessitating the relocation of 133 households. These statistics not only highlight the magnitude of the issue but also the increasing frequency of climate‐related shocks, which have diminished households’ ability to recover between such events and exacerbated both seasonal and chronic food insecurity.

In addition to crop failures, the losses of livestock due to drought have considerably undermined food stability in the region. In 2015, the reported losses encompassed 287 cattle, 97 horses, and 1177 sheep and goats, in addition to the deaths of over 33,000 cattle, 34,681 sheep, and 7461 goats resulting from water and pasture shortages. Livestock play a vital role in rural livelihoods, providing food, income, savings, and a form of insurance against economic shocks. Evidence from comparable African contexts indicates that effective livestock management systems can significantly lower mortality rates and stabilize household incomes amidst climate stress, thus improving food security and resilience [79]. Conversely, in the absence of such systems, livestock losses exacerbate vulnerability and disrupt household food access over time.

The ongoing occurrence of drought‐related shocks underscores the limited adaptive capacity of households and local institutions to cope with climate variability. Studies conducted in dryland and agro‐pastoral systems highlight that insufficient access to institutional support, including early warning systems, extension services, and climate‐responsive infrastructure, severely limits households’ ability to stabilize food supplies [80]. Within the study area, recurrent droughts have diminished coping reserves, resulting in households becoming increasingly reliant on external aid and seasonal coping strategies.

Moreover, the degradation of food stability signifies deeper structural weaknesses within the local food system. Income losses driven by climate change, diminishing livestock resources, and disrupted markets have rendered food availability extremely unpredictable and seasonal. Evidence from regions vulnerable to climate change indicates that achieving long‐term food stability necessitates comprehensive strategies that integrate climate adaptation, diversification of livelihoods, and management of natural resources [81]. Interventions based on landscape approaches, such as water harvesting, small‐scale irrigation, and ecosystem restoration, have demonstrated the potential to enhance resilience when they are aligned with local livelihood requirements and institutional capabilities [82].

Food stability in the examined region has been significantly undermined by the rising frequency and intensity of droughts and the consequent losses in livestock. These climate‐related shocks have shifted food insecurity from a temporary issue to a chronic and ongoing challenge. Therefore, enhancing food stability demands long‐term, coordinated strategies that improve adaptive capacity, safeguard livestock resources, develop climate‐responsive infrastructure, and strengthen institutional support systems to protect households from future climate‐related extremes.

### 4.5. Econometric Analysis

#### 4.5.1. Multicollinearity Estimation

Before proceeding with the estimation of the binary logistic regression model, diagnostic tests were performed to evaluate the existence of multicollinearity among the explanatory variables. This was achieved through the use of variance inflation factors (VIF) and contingency coefficients, as detailed in Appendix Tables A1, A2, and A3. The findings indicated that there was no significant multicollinearity, thereby confirming the appropriateness of the variables for inclusion in the regression model. In line with the work of Gujarati [46], VIF values that fall below the widely recognized threshold of 10 suggest that there is no concerning linear dependence among continuous variables, whereas contingency coefficients that are considerably less than 1 imply weak relationships among categorical variables. The lack of multicollinearity contributes to the reliability and clarity of the estimated coefficients in the subsequent analysis.

#### 4.5.2. Determinants of Food Security Status

A binary logistic regression model was employed to identify the determinants influencing household food security, with food security status regarded as a dichotomous dependent variable. This model included demographic, economic, institutional, and climate‐related factors that may affect household food security outcomes. The adequacy of the model was evaluated through goodness‐of‐fit statistics. The Cox and Snell *R* ^2^ and Nagelkerke *R* ^2^ values were employed to assess the model’s explanatory power. While the Cox and Snell *R* ^2^ is limited to values below one in categorical models, the Nagelkerke *R* ^2^ modifies this restriction to cover the entire range from 0 to 1.

The Nagelkerke *R* ^2^ value of 0.945 (Table 7) signifies a very strong correlation between the explanatory variables and the household food security status. Additionally, the model attained an overall predictive accuracy of 96.7%, demonstrating its effectiveness in accurately classifying both food‐secure and food‐insecure households. The Pearson chi‐square statistic (*χ* ^2^ = 207.205, *p* < 0.001) further substantiates that the model aligns well with the observed data. These findings validate the robustness of the logistic regression model in analyzing the determinants of food security within the study area.

**TABLE 7:** Summary of the binary logistic regression coefficients.

Chi‐square	df	Sig.	−2Log likelihood	Cox and Snell R‐square	Nagelkerke R‐square	Overall correctly prediction
207.205	17	0.000	23.663	0.676	0.945	96.7

Given that the goodness of fit tests validated the model’s adequacy, the analysis moved forward to interpret the results. A positive estimated coefficient in a logistic regression model indicates that the probability of food security increases with a rise in the explanatory variable, provided that other variables are held constant. In contrast, a negative estimated coefficient implies a reduced likelihood of food security as the value of the explanatory variable increases. Eleven variables were descriptively analyzed and recognized as significant at the 1% level, acting as key determinants of food security in the study area, through the application of chi‐squared tests and independent sample *t*‐tests. These variables include age, educational level, size of agricultural holdings, total annual income, livestock ownership, participation in non‐agricultural activities, access to irrigation, access to weather forecasts, access to agricultural extension services, access to credit services, and distance from the market. Furthermore, gender, rainfall, and temperature fluctuations were observed to have a 5% impact on food security.

The results derived from the binary logistic regression analysis shown in Table 8 provide several important insights into the fundamental factors that contribute to food insecurity. None of the variables exhibited a significant effect at *p* < 0.01 (indicating a 99% likelihood). Only four variables showed a significant effect at *p* < 0.05 (representing a 95% probability). The other variables were significant at *p* < 0.1 (indicating a 90% probability), which implies minimal volatility. The negative correlation between age and food security (odds ratio = 0.832) indicates that older individuals are more prone to food insecurity, possibly due to reduced work capacity and difficulties in adapting to changing agricultural practices. This finding aligns with previous studies [83, 84] on the topic of vigilance. In contrast, family size is positively associated with food security (odds ratio = 3.607), as larger families gain from increased labor for agricultural activities and improved access to food resources. This finding contradicts earlier studies [47, 83] that suggested larger households may be more vulnerable to food insecurity due to elevated consumption demands.

**TABLE 8:** Results of binary logistic regression model variables.

Explanatory variables	*β*	SE	*p*‐value	Exp(*β*)
Sex	1.164	2.243	0.604	3.203
Age	−0.196	0.118	0.095^∗^	0.832
Family size	1.283	0.716	0.073^∗^	3.607
Educational status	4.226	2.187	0.053^∗^	1.015
Households engaged in nonfarm	−3.084	2.417	0.202	0.046
Livestock ownership in TLU	1.501	0.675	0.026^∗∗^	4.487
Land size (ha)	1.368	2.607	0.600	3.927
Use of irrigable land	−2.934	1.532	0.055^∗^	0.053
Total annual income	0.001	0.000	0.028^∗∗^	1.001
Changes in temperature	6.626	4.722	0.161	754.69
Changes in rainfall	−11.801	5.884	0.045^∗^	0.0001
Noticed changes in drought frequency	−11.870	5.652	0.036^∗∗^	0.0001
Noticed changes in flood frequency	−1.604	10,784	0.369	0.201
Use credit services	−4.527	2.617	0.084^∗^	0.011
Access to agricultural extension services	2.806	3.513	0.424	16.548
Access to the weather forecasts	2.352	2.509	0.349	10.505
Distance from market	−2.114	1.759	0.229	0.121
Constant	−11.865	7.754	0.126	0.0001

*Note:* β = estimated coefficient, *p*‐value = significance level, Exp(β) = odds ratio.

Abbreviation: SE = standard error.

^∗^Significant at 10%.

^∗∗^Indicates significant at 5%.

Furthermore, attaining a higher level of education (odds ratio = 1.015) significantly increases the probability of achieving food security, as households with educated members are more inclined to implement modern agricultural practices and obtain information on climate resilience strategies. This finding aligns with earlier studies, such as [85], which have consistently shown a negative relationship between educational attainment and poverty levels. These studies suggest that higher education enables households to adopt more efficient farming methods, gain improved access to agricultural innovations, and enhance their ability to tackle challenges such as climate change, thereby reducing the risk of food insecurity. In addition, the ownership of livestock plays a vital role in ensuring food security, with a probability ratio of 4.487 indicating that households with a greater number of livestock are better positioned to cope with food insecurity. Livestock not only provides a source of income but also acts as a safety net during difficult times, serving as a form of savings.

Interestingly, there is a negative relationship between access to irrigated land and food security (odds ratio = 0.053). This may be attributed to the high costs associated with irrigation systems and challenges related to water scarcity, which could exacerbate food insecurity. These challenges significantly contribute to food insecurity and support the conclusions of previous studies [45, 86, 87]. On the other hand, total annual income emerges as another essential factor positively linked to food security (odds ratio = 1.001), suggesting that a higher income level increases the likelihood of food security by enhancing purchasing power and access to agricultural resources. These results are consistent with previous studies [45, 86–88].

### 4.6. Adaptation Strategies

Households within the study region utilize a range of adaptation strategies to address climate variability and weather‐related events. These strategies, which are frequently employed in conjunction [89], encompass altering planting schedules, selecting drought‐resistant crop varieties, increasing fertilizer application, implementing soil and water conservation techniques, and diversifying income through noncrop activities. In total, 82.1% of households indicated that they have adopted at least one adaptation measure, with 98.3% of food‐secure households and 74.4% of food‐insecure households engaging in adaptation practices (Table 9). A chi‐squared test (*χ* ^2^ = 15.562, *p* < 0.001) demonstrated a significant correlation between adaptation measures and food security, indicating that adaptation initiatives contribute positively to food security.

**TABLE 9:** Households’ responses to adaptation actions in relation to food security status.

Variable	Response	Food‐secure HH (%)	Food‐insecure HH (%)	Total HH (%)	Chi‐square test
Adaptation action taken by households	Yes	58 (98.3%)	93 (74.4%)	151 (82.1%)	*x* ^2^ = 15.562 df = 1
	No	1 (1.7%)	32 (25.6%)	33 (17.9%)	*p* < 0.001

The main strategies for adaptation include practices associated with crop and soil management, along with the diversification of livelihoods. Key strategies for crop management, as demonstrated in Table 10, involved changing the planting date (85.3%), using improved varieties (78.8%), increasing fertilizer application (62.5%), and adopting drought‐resistant crops (39.7%). The primary areas of focus were soil and water management (86.4%), energy harvesting (20.1%), and tree planting (35.3%). Furthermore, 21% of households reported a transition toward non‐agricultural activities, such as retail and handicrafts. The most widely endorsed strategies for addressing climate change consist of adjusting planting dates and applying soil and water management practices.

**TABLE 10:** The adaptation actions taken by households.

Adaptation strategies	Applied (%)	Not applied (%)
Using improved crop and livestock varieties	145 (78.8%)	39 (21.2%)
Changing planting dates	157 (85.3%)	27 (14.7%)
Adopting drought‐resistant crops	73 (39.7%)	111 (60.3%)
Soil and water conservation techniques	159 (86.4%)	25 (13.6%)
Diversification into off‐farm activities	37 (20.1%)	147 (79.9%)
Water harvesting	37 (20.1%)	147 (79.9%)
Planting trees	65 (35.3%)	119 (64.9%)
Improved use of irrigation	37 (20.1%)	147 (79.9%)
Improved use of fertilizer	115 (62.5%)	69 (37.5%)

## 5. Conclusion and Recommendations

Food insecurity represents a significant and urgent challenge in Enebse Sar Midir, where more than two‐thirds of households face inadequate food availability, access, or utilization. Critical demographic and socioeconomic elements, including education, land size, livestock ownership, and income, have shown a positive relationship with food security. Conversely, the vulnerability of older individuals and households impacted by unpredictable rainfall and limited adaptation resources has escalated. The strong link between household perceptions and meteorological data confirms that climate change is not merely a scientific reality but also a lived experience in rural areas. The rising frequency of droughts and floods, along with increasing temperatures and diminishing rainy seasons, has profoundly impacted agricultural productivity, leading to both chronic and seasonal food shortages. Despite widespread awareness of these issues and observable adaptation initiatives, the persistent lack of financial, technical, and institutional resources continues to hinder many households from achieving food self‐sufficiency.

The logistic regression analysis has also revealed that the security of the food supply is significantly influenced by both environmental factors and human capital. Ownership of livestock, levels of income, and education have been identified as strong indicators of resilience, while the adverse effects of climate variability underscore the pressing necessity for targeted interventions. Adaptation strategies, including soil and water conservation, adjustments in planting dates, and the adoption of improved crop varieties, have been increasingly implemented in households engaged in food production, emphasizing their critical role in alleviating climate vulnerability. In light of these findings, the following policy recommendations are suggested to enhance food security and resilience in the study area—expand access to drought‐resistant crops, encourage water‐efficient agricultural practices, enhance availability and utilization of both organic and chemical fertilizers, and bolster farmer education in climate‐smart agricultural techniques.

## Author Contributions

Kiros Getachew Belachew, Berhanu Tadesse Beyene, and Balew Yibeltal Bezabih equally contributed to the conceptualization of the study, methodology design, data collection, and analysis. They facilitated the data collection process and entered the data into Excel and SPSS for analysis. Additionally, they were responsible for drafting the manuscript, addressing reviewer feedback, and refining the grammar and overall quality of the manuscript through editing and revisions.

## Funding

There are no funding or other issues with this manuscript.

## Ethics Statement

This research adhered to ethical guidelines approved by the Department of Natural Resource Management (approval number 2024/NARM/06) within the College of Agriculture and Natural Resources at DMU. All participants were fully informed about the study’s objectives and voluntarily consented to participate. Written informed consent was obtained from all participants. To ensure privacy, participant identities were kept anonymous.

## Conflicts of Interest

The authors declare no conflicts of interest.

## Data Availability

The data supporting this study are available with the researcher and will be shared upon request.

## Appendix A Operational Variables

**TABLE A1:** Showing VIF test result.

Independent variables	Coefficients	
	Tolerance	VIF
Age	0.748	0.337
Family size	0.949	1.054
Livestock ownership (TLU)	0.443	2.259
Land size	0.421	2.373
Total annual income	0.292	3.422

*Note:* Dependent variable: food secure and food insecure.

**TABLE A2:** Showing contingency coefficients for the dummy.

	1	2	3	4	5	6	7	8	9	10	11	12
Sex	1	0.149	0.076	−0.002	0.216	0.003	−0.008	−0.170	0.293	0.127	−0.065	0.147
Distance from market		1	−0.043	0.053	0.375	−0.108	0.004	−0.160	0.039	0.102	0.258	−0.071
Educational status			1	−0.075	0.281	−0.212	0.028	0.157	0.177	−0.063	0.056	−0.200
Engaged on off‐farm				1	0.213	0.217	−0.169	−0.010	−0.104	0.431	0.084	−0.375
Access to irrigable land					1	−0.023	−0.100	0.106	0.313	0.306	0.116	−0.321
Changes in temperature						1	−0.351	−0.295	0.106	0.199	−0.091	−0.089
Changes in rainfall							1	−0.227	0.033	0.015	−0.343	0.250
Changes in drought frequency								1	−0.071	−0.203	0.169	−0.056
Changes in flood frequency									1	0.267	−0.157	0.100
Access to credit service										1	−0.389	−0.167
Access to an agree extension											1	−0.310
Access to weather forecasts												1

**TABLE A3:** TLU (tropical livestock unit) conversion factors.

Name of animals	Conversion factors
Ox/bull	1.2
Cow	1
Heifer	0.6
Calf	0.3
Sheep/goat	0.17
Donkey	0.65
Horse	0.8
Mule	0.7
Chicken	0.01

*Note:* Source: [90].

## Appendix B Metrological Data From the Study Area (1991–2020)

**TABLE A4:** Monthly rainfall and temperature variability (1991–2020).

Climate elements	Descriptive statistics	Jan	Feb	Mar	April	May	Jun	Jul	Aug	Sep	Oct	Nov	Dec
Rainfall	Mean	13.7	17.2	41.6	61.8	73.0	116.0	282.9	246.4	114.1	55.9	20.9	7.9
	CV	1.91	1.36	0.79	0.71	0.70	0.40	0.31	0.32	0.48	0.92	0.96	1.62

Temperature	Mean	23.8	24.0	24.3	24.5	24.3	23.2	22.0	22.3	23.1	23.4	23.7	23.5
	CV	0.043	0.040	0.047	0.039	0.037	0.058	0.061	0.065	0.056	0.038	0.043	0.049
